# Appropriate and inappropriate methods for investigating the “gateway” hypothesis, with a review of the evidence linking prior snus use to later cigarette smoking

**DOI:** 10.1186/s12954-015-0040-7

**Published:** 2015-03-20

**Authors:** Peter N Lee

**Affiliations:** P.N.Lee Statistics and Computing Ltd, 17 Cedar Road, Sutton, Surrey, SM2 5DA UK

**Keywords:** Gateway, Snus, E-cigarettes, Smoking, Study design

## Abstract

**Background:**

The “gateway hypothesis” usually refers to the possibility that the taking up of habit A, which is considered harmless (or less harmful), may lead to the subsequent taking up of another habit, B, which is considered harmful (or more harmful).

**Methods:**

Possible approaches to designing and analysing studies to test the hypothesis are discussed. Evidence relating to the use of snus (A) as a gateway for smoking (B) is then evaluated in detail.

**Results—design and analysis considerations:**

The importance of having appropriate data available on the sequence of use of A and B and on other potential confounding factors that may lead to the taking up of B is emphasised. Where randomised trials are impractical, the preferred designs include the prospective cohort study in which ever use of A and of B is recorded at regular intervals, and the cross-sectional survey in which time of starting to use A and B is recorded. Both approaches allow time-stratified analytical methods to be used, in which, in each time period, risk of initiating B among never users of B at the start of the interval is compared according to prior use of A. Adjustment in analysis for the potential confounding factors is essential.

**Results—review of evidence:**

Of 11 studies of possible relevance conducted in Sweden, Finland or Norway, only one seriously addresses potential confounding by those other factors involved in the initiation of smoking. Furthermore, 5 of the 11 studies are of a design that does not allow proper testing of the gateway hypothesis for various reasons, and the analysis is unsatisfactory, sometimes seriously, in all the remaining six.

**Conclusions:**

While better analyses could be attempted for some of the six studies identified as having appropriate design, the issues of confounding remain, and more studies are clearly needed. To obtain a rapid answer, a properly designed cross-sectional survey is recommended.

## Background

In the context of drugs, the “gateway hypothesis” predicts that the use of less deleterious drugs can lead to a future risk of using more dangerous hard drugs or crime. The hypothesis has recently been discussed in detail by Vanyukov et al. [1]. He points out that an earlier “stepping-stone” theory first appeared in the 1930s which “assumed that consumption of a soft drug such as marijuana inexorably sets an individual on a trajectory to addiction to hard drugs” but notes that when the gateway hypothesis was first advanced by Kandel about 40 years ago [2], the inevitability assumption was relaxed. The concern here is simply with testing whether use of the first product makes it more likely, not necessarily inevitable, that the second product will later be used. From the point of view of Kandel and Kandel [3], the causal agent is the drug itself and how it “primes” the brain to react to later drug use.

The term has also been used in the context of cigarette smoking, with concern that the prior use of Swedish moist snuff (snus, known to have only minor health effects on health [4,5]) or of electronic cigarettes (E-cigarettes, assumed from their design to have little effect on health) might increase the probability of initiating the smoking of conventional cigarettes (known to have many harmful effects [6]).

The main body of this paper is divided into two, linked, parts. The first “Investigating the gateway hypothesis—difficulties and recommended methods” considers in general terms how the gateway hypothesis should be investigated and discusses the appropriate study designs and methods that should be used and the various dangers of bias.

The second part “Does snus use act as a gateway to cigarette smoking?” considers those studies that appear to provide data relevant to the relationship between snus and conventional cigarette smoking (regardless of whether the publications were specifically intended to address the gateway hypothesis), assesses how much they contribute to a valid test of the gateway hypothesis in the light of the methods considered appropriate in the previous part, discusses the need for additional evidence, and draws conclusions relating to the question posed. It is intended to be just one example of how evidence relating to a possible gateway should be evaluated.

It should be noted that attention is limited to the gateway hypothesis, as usually defined, which is only concerned with exposures leading to uptake of the more dangerous substance. While some may consider events leading to giving up the dangerous substance as also being relevant, distinguishing “gateway in” and “gateway out”, this is not the usual interpretation of the gateway hypothesis. Thus, for example, data relevant to whether taking up snus makes a smoker more likely to quit are not considered here, as being concerned with answering a different (though also relevant) question.

## Investigating the gateway hypothesis—difficulties and recommended methods

### Terminology

For convenience, we refer to the agent that may have the gateway effect as A and the adverse event it may lead to as B. In the present context, A represents snus or E-cigs, while B represents smoking conventional cigarettes, but the principles described are generalisable. It should also be noted that the reader should assume that any future reference in this paper to “cigarettes” implies conventional cigarettes, unless otherwise stated.

### Evidence of an association may not indicate a causal relationship

Observing an association between the use of A and the use of B does not, of itself, confirm the gateway hypothesis. An association may arise, in theory, for one of three reasons:

- A causes B, i.e. the gateway hypothesis is true
- B causes A, i.e. reverse causation
- a third factor (or set of factors) C causes both A and B, i.e. confounding

### Reverse causation

Whether the possibility of reverse causation (B causes A) is a major concern depends upon the context. For example, no one has suggested that the strong association between smoking and lung cancer is due to presence of the cancer prompting the sufferer to take up smoking, a possibility that is in any case implausible as smoking typically starts before age 30 and lung cancer mainly occurs after age 60. Similarly, reverse causation would not be an important issue in any situation where taking up A usually occurs in childhood, while taking up B usually occurs in adulthood.

However, where A represents snus or E-cigarettes, and B is conventional cigarettes, reverse causation can certainly not be ruled out. In this scenario, it is essential to take the sequence of use into account since many cigarette smokers start smoking before they start to use snus or E-cigarettes. This implies that studies which only collect data on use of A or of B relating to one time point are of no value in testing the gateway hypothesis.

An appropriate study design to avoid issues of reverse causation is a prospective cohort study based on subjects who, at baseline, have never used B, but who may or may not have ever used A, with information on subsequent use of B recorded.

In the simplest situation, use of B is only recorded at a single follow-up interview, with the relative risk (RR) and 95% confidence interval (CI) of using B at follow-up according to ever use of A at baseline estimated by standard methods [7,8]. Here, the best endpoint to quantify use of B would be ever use by the time of follow-up. However, an analysis based on current use of B at follow-up would also provide some relevant information, though this endpoint would be inferior as those who started and then stopped using B in the interval would not be distinguished from those that had never used B.

Where data are collected at multiple follow-up interviews on ever (or current) use of B, an improved analysis can be conducted. Here, RRs (95% CIs) are estimated for successive time periods and combined using standard RR methods for data stratified by time of follow-up [7,9]. Here, the at-risk population for each time period includes only those subjects who have never used B up to the beginning of that period, with subjects contributing to the analysis only until the time period during which they started to use B.

This method of analysis can also be used where data are collected on time of starting to use B, though if such data are collected at the end of follow-up, problems may arise from inaccurate recall, and there is a possibility of recall bias if, for example, current habits affect reporting of past habits.

Note that, in all these analyses, alternative definitions of “use” are possible, corresponding to any use at all, or to some defined level of occasional or regular use. While it might be regarded as unlikely for biological and psychological reasons that trying A on a single occasion might affect the uptake of B some years later, the argument that longer-term use of A might be a gateway to B seems more plausible.

One can also conduct similar analyses based on a cross-sectional survey in which data are recorded for each subject on the time of uptake of A and B, though again this is subject to errors in recall and the possibility of recall bias. Here, a data set exactly equivalent to that obtained from a prospective cohort study can readily be constructed and the same statistical methods used. Constructing a data set retrospectively may lead to lack of representativeness, if a proportion of the initial population of interest die by the time the survey takes place. Since few people take up smoking much after age 25, and since most “gateway” studies are carried out in schoolchildren and young adults where mortality rates are low, lack of representativeness of survivor populations should not be a material issue. Indeed, lack of representativeness may be less of a problem for analyses based on cross-sectional studies than it is for prospective cohort studies with substantial loss to follow-up, especially if the reasons for dropout are correlated with use of A or B. Loss to follow-up may particularly be problematic where B represents hard drugs or crime.

It should also be noted that analysis based on a cross-sectional survey in which data are recorded only on the order of uptake of A and B cannot provide an unbiased assessment of the gateway hypothesis, due to failure to adjust for the time available in which to start uptake of B. As shown in Table 1, and as has been previously demonstrated [4], it is possible to derive substantially reduced ORs relating uptake of smoking to prior snus use by following a hypothetical sample of never tobacco users, with the rates of initiation of smoking and of snus assumed to be independent (RR = 1) and with the onset time distributions for both products assumed to be the same. The bias arises because the time available for initiation is not controlled for in the analysis, those who have never used snus being able to initiate smoking at any age, while those who used snus before they started to smoke being able only to initiate smoking from the age they started using snus.

**Table 1:** **Bias in estimating the association between uptake of B and prior use of A**

	**Neither (i)**	**A only (ii)**	**B only (iii)**	**Both – A first (iv)**	**Both – B first (v)**
Time 0	10000	0	0	0	0
Start using A	*250 i → ii*				
Start using B	*500 i → iii*				
Time 1	10000 − 250 − 500 = 9250	250	500	0	0
Start using A	*231 i → ii*		*13 iii → v*		
Start using B	*463 i → iii*	*13 ii → iv*			
Time 2	9250 − 231 − 463 = 8556	250 + 231 − 13 = 469	500 + 463 − 13 = 950	13	13
Start using A	*214 i → ii*		*24 iii → v*		
Start using B	*428 i → iii*	*23 ii → iv*			
Time 3	8556 − 214 − 428 = 7915	469 + 214 − 23 = 659	950 + 428 − 24 = 1354	13 + 23 = 36	13 + 24 = 36
Start using A	*198 i → ii*		*34 iii → v*		
Start using B	*396 i → iii*	*33 ii → iv*			
Time 4	7915 − 198 − 396 = 7321	659 + 198 − 33 = 824	1354 + 396 − 34 = 1716	36 + 33 = 69	36 + 34 = 70
P1: Proportion who start B among A first users			69/(824 + 69)		=0.08
P2: Proportion who start B among others			(1716 + 70)/(7321 + 1716 + 70)		=0.20
P3: Proportion who start B among never A users			1716/(7321 + 1716)		=0.19
OR1 as defined in (using P1 and P2)			(0.08/(1-0.08))/(0.20/(1-0.20))		=0.34
OR2 as defined in (using P1 and P3)			(0.08/(1-0.08))/(0.19/(1-0.19))		=0.36
*RR stratified by period* (*using columns i and ii*)			Period 1–2	(13/250)/(463/9250)	=1
			Period 2–3	(23/469)/(428/8556)	=1
			Period 3–4	(33/659)/(396/7915)	=1

The example involves four time periods, with starting of A and B assumed to be independent. Rows in normal type style indicate time points, and rows in italicized type style indicate the periods between the time points. In each period, 2.5% of those who have never used A by the previous time point start A, and 5% of those who have never used B by the previous time point start B. The arrows and roman numerals indicate the column to which starters are re-allocated at the next time point. It is assumed that no individual starts both products in a single period. The values are shown rounded to nearest whole numbers but are calculated to full precision (so that there are some apparent discrepancies in the additions).

Case-control designs are often used in epidemiological studies where B is a rare event (e.g. lung cancer). However, where B is a common event (e.g. cigarette smoking), there would seem to be no obvious point in using such a design, though one could do so, with say cases = conventional cigarette smokers and controls = never smokers.

### Confounding

While the issue of reverse causation can quite easily be got round by a suitable study design, the problem of confounding (C causes A and B) cannot. It is a real possibility that characteristics of the subject might affect the chance of trying or taking up A or B. Such characteristics might include genetic factors, attitudes to tobacco developed from education or the media, personality and availability of money.

The bias due to this can be illustrated by a hypothetical example in which 50% of the population, who we might term “unadventurous”, would never consider using A or B, and among the other 50%, “risk takers”, 40% use A and 40% use B, with the use of A and of B assumed to be independent. As shown in Table 2, in a large sample of the total population, one would expect to have approximately 8% dual users, 12% users of A only, 12% users of B only and 68% users of neither. Here, the expected value of the unadjusted odds of using B given use of A (800/1,200 = 0.667) is substantially higher than that of using B given no use of A (1,200/6,800 = 0.176), with the odds ratio (OR) equal to 3.78. However, since the data are generated under the assumption that use of A does not actually affect use of B, this elevated odds ratio must in fact be due to confounding. If it were possible to identify the unadventurous and remove them from the analysis, the confounding effect would vanish as the odds of using B given no use of A would then be 1,200/1,800 (=0.667), the same as that for the odds of using B given use of A.

**Table 2:** **First example of confounding**

Unadventurous				Risk takers				Overall			
5000				5000				10000			
*No A users*				*40% use A*				(Sum over unadventurous and risk takers)			
↙		↘		↙		↘		↙		↘	
A		Not A		A		Not A		A		Not A	
0		5000		2000		3000		2000		8000	
*No B users*		*No B users*		*40% use B*		*40% use B*		(sum)		(sum)	
↙	↘	↙	↘	↙	↘	↙	↘	↙	↘	↙	↘
B	Not B	B	Not B	B	Not B	B	Not B	B	Not B	B	Not B
0	0	0	5000	800	1200	1200	1800	800	1200	1200	6800
				OR (within risk takers) = (800/1200)/(1200/1800) =1.00				OR (overall, unadjusted) = (1000/1200)/(1200/6800) = 3.78			

It is assumed that there is no use of A or B among the “unadventurous”, and that use of A and B is independent among the ‘risk takers’. The table shows the joint distribution of use of A and B, separately for the “unadventurous” and the “risk takers” and overall, and demonstrates that the unadjusted OR shows a markedly spurious relationship.

Failure to adjust for this unadventurous/risk takers factor will also lead to overestimation of the true odds ratio where a true association actually exists. Thus, consider a second hypothetical example starting from the same situation of 50% unadventurous and 50% risk takers, where, in the risk takers, 40% use A, with use of B 50% given A and 30% given no A. As shown in Table 3, the expected distribution would be 10% dual users, 10% users of A only, 9% users of B only and 71% users of neither. The OR calculated within the risk takers would then be 2.33, but the unadjusted OR within the whole population would be 7.89.

**Table 3:** **Second example of confounding**

Unadventurous				Risk takers				Overall			
5000				5000				10000			
*No A users*				*40% use A*				(Sum over unadventurous and risk takers)			
↙		↘		↙		↘		↙		↘	
A		Not A		A		Not A		A		Not A	
0		5000		2000		3000		2200		8000	
*No B users*		*No B users*		*50% use B*		*30% use B*		(sum)		(sum)	
↙	↘	↙	↘	↙	↘	↙	↘	↙	↘	↙	↘
B	Not B	B	Not B	B	Not B	B	Not B	B	Not B	B	Not B
0	0	0	5000	1000	1000	900	2100	1000	1000	900	7100
				OR (within risk takers) = (1000/1000)/(900/2100) = 2.33				OR (overall, unadjusted) = (1000/1000)/(900/7100) = 7.89			

As in Table 2, it is assumed that there is no use of A or B among the “unadventurous”. Here, however, the use of A and B is assumed to be correlated among the “risk takers”. It is demonstrated that the unadjusted odds ratio is substantially higher than the OR in the “risk takers”.

In the two examples above, it has been assumed that the unadventurous do not take up A or B in any circumstances. The same conclusion that failure to adjust for the factor would overstate the association, would also apply if this assumption were weakened, with the unadventurous just being much less likely to take up B. Suppose that, in a third example, we assume that, in the unadventurous, 4% use A, with use of B 5% given A and 3% given no A, with use of A and B in the risk takers the same as in the second example. Here (Table 4), the expected distributions in the unadventurous are 0.2% dual users, 3.80% users of A only, 2.88% users of B only and 93.12% users of neither, with the corresponding distributions in the risk takers being 20%, 20%, 18% and 42%. Here, the ORs are 1.70 for the unadventurous and 2.33 for the adventurous, yielding a combined adjusted OR of 2.31. However, the unadjusted OR based on the combined distributions of 10.1%, 11.9%, 10.44% and 67.56% is much higher, at 5.49.

**Table 4:** **Third example of confounding**

Unadventurous				Risk takers				Overall			
5000				5000				10000			
*4% use A*				*40% use A*				(Sum over unadventurous and risk takers)			
↙		↘		↙		↘		↙		↘	
A		Not A		A		Not A		A		Not A	
200		4800		2000		3000		2200		7800	
*5% use B*		*3% use B*		*50% use B*		*30% use B*		(sum)		(sum)	
↙	↘	↙	↘	↙	↘	↙	↘	↙	↘	↙	↘
B	Not B	B	Not B	B	Not B	B	Not B	B	Not B	B	Not B
10	190	144	4656	1000	1000	900	2100	1010	1190	1044	6756
OR (within unadventurous) = (10/190)/(144/4656) = 1.70				OR (within risk takers) = (1000/1000)/(900/2100) = 2.33				OR (overall, unadjusted) = (1010/1196)/(1044/6756) = 5.49			
OR (overall, adjusted for risk taking) = ((10 × 4656)/5000 + (1000 × 2100)/5000)/((190 × 144)/5000 + (1000 × 900)/5000) = 2.31											

In this example, unlike in Tables 2 and 3, there is some use of A and B in the “unadventurous”, though much less than in the “risk takers”. It is also assumed that the use of A and B is correlated in both subgroups. It is demonstrated that the unadjusted OR substantially overestimates the adjusted OR.

How do we remove bias due to confounding? In principle, the best approach is to conduct a large randomised trial, in which those who have never used B are randomly assigned to use or not use A. However, even if this were restricted to volunteers who would be willing to use A, if assigned to do so, it would be regarded as unethical. An alternative type of trial using some form of randomisation might involve introducing A into the market in randomly selected areas in a country and following trends in B. This would be analogous to the study conducted by Rose and Hamilton in 1983 [10], in which mortality from various causes was compared in factories randomly assigned either to receive or not to receive anti-smoking advice. However, unless the number of randomization units (factories in the Rose and Hamilton example) is large, there is no guarantee that the two groups being compared are similar in respect of relevant confounders. Such a design would probably be impractical.

Given that a randomised approach does not seem possible, all that remains is an approach where one collects information on relevant confounders (C) and adjusts for them in analysis. If an association between A and B exists and is little affected when analysed taking into account what is considered a reasonably complete list of relevant confounders, then one would accept the gateway hypothesis. If, on the other hand, it is eliminated by adjustment, one would reject the hypothesis, explaining the unadjusted association by confounding. What might well happen in practice is that adjustment considerably reduces the association but does not totally eliminate it. Here, one may also tend to reject the hypothesis and assign the adjusted association to “residual confounding”, especially where one cannot readily exclude the existence of a relevant confounding factor that has not been accounted for or where there are inaccuracies in determining existing confounding variables.

To illustrate residual confounding due to inaccuracies in the confounding variable, consider a further hypothetical example, similar to the first example, where instead of the unadventurous and risk taker groups being accurately known, 20% of the unadventurous are misclassified as being risk takers by some procedure. Here, instead of the expected OR calculated within the risk takers being (800/1,200)/(1,200/1,800) = 1 indicating a lack of relationship, as assumed, the expected OR would become (800/1,200)/(1,200/[1,800 + 1,000]) = 1.56. The issue of residual confounding has been considered in various publications. For example, Tzonou et al. [11], in a detailed investigation with extensive tabulations, concluded that “even misclassification rates of 10% can prevent adequate control of confounding” and emphasised the difficulties of drawing inferences about weak associations in the presence of variables which have a strong effect.

Illustrations of the potential importance of confounding in the tobacco area can be found in US studies of the relationship of smokeless tobacco use to subsequent initiation of smoking. Thus, in a study by Severson et al. [12] in which boys aged 12–13 were followed up for 2 years, and smoking after 2 years was related to smokeless tobacco use at baseline among 2,509 never smokers, the unadjusted OR of 4.25 (95% CI 3.02–5.99), calculated from data presented, was reduced to 2.62 (1.31–5.22) after adjustment for six predictors of smoking initiation (parent, sibling or close friend smoking; low academic grades; alcohol use in the last 30 days; and an index of deviant behaviour). Furthermore, a study by Timberlake et al. [13], based on 496 current smokeless tobacco users and 496 non users matched on a “propensity score” who were followed from adolescence into young adulthood, found no significant evidence of an association of smokeless tobacco use with the risk of smoking. Whereas elevated odds ratios were seen in the unadjusted analyses, odds ratios were close to 1 in all the adjusted analyses whether the period of follow-up was 1 or 6 years, the restriction was to never smokers or to nonsmokers, and whether the outcome was ever daily smoking or amount smoked. The “propensity score” was based on a range of variables measured at baseline, including demographic variables, smoking by friends or parents, behavioural risk variables (e.g. binge drinking, use of cannabis, motor cycling), a measure of delinquency and a measure of depressive symptoms.

The approach used here is a standard epidemiological one in which causality can be asserted if the use of B follows use of A in time in an analysis in which all confounding factors are fully accounted for. The problem of course is ensuring full accounting, but if the association is a strong one and remains strong after reasonable confounder adjustment, then causality can, for practical purposes, be assumed. Such is the situation in regard to many health effects regarded as causal, smoking and lung cancer, for example. Demonstrating causality is more dubious when the association is weak and is reduced but not completely eliminated by adjustment. While this is unsatisfactory, alternative approaches, such as using the counterfactual framework [14,15] or instrumental variables [16], seem unlikely to clarify the existence of a causal relationship between use of A and subsequent use of B.

## Does snus use act as a gateway to cigarette smoking?

### Methods

Publications describing studies in Sweden, Norway and Finland relevant to snus use as a possible gateway to the use of cigarettes were obtained using the methods described in 2011 for a review of the evidence relating snus to health [4]. The searches used for that review were updated by further searches on MEDLINE (from January 1st, 2011), from the files used for the regularly updated International Smoking Statistics [17], from in-house files on snus and from citations in recent reviews and in papers obtained. Studies which only provided data on the joint relationship of snus and cigarette smoking at one time point were not considered, though as will become evident, studies providing an inappropriate test of the gateway hypothesis were considered.

### Results

The sections that follow assess in turn the individual studies identified.

### Survey of living conditions (ULF) (Sweden)

In 2009, Stenbeck et al. [18] described a series of analyses based on 2,156 Swedish men, aged 16 to 84 years in 1988–1989, who responded to questions about tobacco habits then, and eight years later in 1996–1997. The authors concluded that “snus contributed to the reduction of smoking among Swedish males in the 1990s”, considering that though snus contributed to the initiation of smoking, it contributed more to its cessation. Some points should be noted about this publication. First, the data analysed relate only to current rather than ever use at each time point. Thus, some non-smoking snus users in 1988–1989 may have smoked earlier, possibly before they started to use snus. Second, the results presented make no adjustment for potential confounding variables; analyses adjusted for socioeconomic status are mentioned but not reported as no confounding effects or interaction was found. Third, no table was presented showing the numbers of men by tobacco use at the two time points or giving RRs of becoming smokers by snus use at baseline. However, Dr Stenbeck kindly provided the necessary table which allowed calculation of RRs (95% CIs) separately for the two age groups considered, 16–44 and 45–84 years. These were 1.38 (0.68–2.83) in the 16–44 age group, based on 31 initiators (nonsmokers who became smokers at the follow-up interview), and 2.47 (0.98–6.18) in the 45–84 age group, based on 22 initiators.

Earlier, in 1996, a paper by Tillgren et al. [19], mainly concerned with tobacco cessation, provided results on 2,383 men aged 16–84 who provided data on current snus use and smoking in 1980–1981 and in 1988–1989. Using the joint distribution of habits given in a figure in that paper, an RR (95% CI) of 1.69 (1.13–2.54) can be calculated, based on 109 initiators. The main weaknesses of these analyses are that they do not provide data for those who have never smoked at baseline, and that RRs adjusted for any potential confounding variables cannot be calculated.

### Children’s smoking and environment in Stockholm County (BROMS) (Sweden)

In 2008, Galanti et al. [20] described the results of analyses linking early trying of cigarettes and snus to subsequent taking up of the habits, based on a study which followed 2,938 Swedish adolescents from grade 5 in 1998 (age 11–12) through to grade 9 (the end of compulsory school education) and subsequently to the end of follow-up 3 years later at about age 18. A complete assessment of tobacco use was conducted at almost every year, which included information on ever and current use of cigarettes and snus. Results are presented comparing current smoking at end of follow-up by ever use of tobacco at baseline. Of those 26 adolescents who reported having used snus only at baseline, 13 (50%) reported current smoking at the end of follow-up. In contrast, current smoking was reported in 424 of 1,536 (27.6%) who reported never using tobacco (either snus or smoking) at baseline. Based on these data, an unadjusted RR (95% CI) of 1.54 (0.98–2.42) can be calculated, while the authors reported an OR, adjusted for sex and age at entry, of 1.95 (0.96–3.80).

Increased unadjusted RRs (95% CIs) of 2.28 (1.50–3.48) in boys and 1.65 (0.77–3.50) in girls could also be calculated from an earlier paper by Galanti et al. [21] based on the first year’s follow-up.

The problems with these analyses relate to the lack of adjustment for other potential predictors of uptake of smoking and in the 2008 paper [20] of failure to carry out an improved analysis based on the complete tobacco use history obtained, combining information on initiation of smoking within each follow-up period. It is also not apparent why the authors presented results as ORs rather than as RRs.

The 2008 paper [20] also presents results comparing the odds of current smoking at end of follow-up according to whether snus was used first; cigarettes were used first or both were started at the same time. Although the odds was lowest if snus was used first, even if adjustment was made for sex and the age of initiation of tobacco, these analyses do not provide any valid test of the gateway hypothesis. The authors concluded in 2008 [20] that “Progression of tobacco use in adolescence is not predicted by onset with snus or cigarettes, but rather by initiation with both tobacco types close in time and/or at young age. The proportion of adolescent smoking prevalence attributable to a potential induction effect of snus is likely small”. This contrasts with their earlier conclusion [21] that “In most cases, experimentation with oral snuff among boys marks the transition to cigarette smoking”.

### Spanning across lifespan twin study (SALT) (Sweden)

In 2005, Furberg et al. [22] presented analyses based on 14,424 male twins born in Sweden before 1959 who were interviewed by telephone and provided detailed data on tobacco use, including age at first use (Some of these data were also presented in a later paper [23] which did not specifically test the gateway hypothesis.). To investigate the gateway hypothesis, they compared men who used snus before they started to smoke to men who never used snus in relation to any lifetime smoking. Finding that lifetime smoking was negatively related, after adjustment for age, to both regular snus use (OR 0.2, 95% CI 0.2–0.3) and occasional snus use (OR 0.5, 95% CI 0.3–0.7), Furberg et al. [22] concluded that snus use was not associated with smoking initiation. However, the test used is invalid as discussed above (see Table 1).

### Your country and your life survey (Sweden)

In 2006, Ramström and Foulds [24] described the results of a cross-sectional survey of adult Swedes in 2001–2002 in which detailed data were collected on time of initiating daily smoking and snus, from which they concluded that “use of snus in Sweden is associated with a reduced risk of becoming a daily smoker”. Among 502 men who started tobacco use as snus use, 100 (20%) subsequently smoked cigarettes, while among the 2,623 men who had not used snus before, 1,226 (47%) did so. The OR was estimated as 0.28 (95% CI 0.22–0.36). Though this was unadjusted for age or any other factor, the authors state that it remained similar and still significant, across age groups and levels of education. As for the study by Furberg et al. [22] considered in the previous section, the test used is invalid, because it fails to control for the time available for initiation of smoking (see also Table 1). Both studies appear to have the data available to do an improved analysis, though the extent to which they would be able to control for other factors predictive of initiation of smoking may be limited.

### Västerbotten intervention programme (VIP) (Sweden)

In this study in a county in Northern Sweden, which started in 1985, all adults were invited to health examinations when they reached the ages 30 (until 1995 only), 40, 50 or 60 years. At baseline and follow-up, the subjects reported whether they were current, former or never smokers (with smokers reporting regular or occasional use) and whether they were current, former or never regular snus users.

In 2009, Lundqvist et al. [25] reported results of follow-up 10 years later in 16,486 participants examined initially in 1990–1994. No specific test of the gateway hypothesis was conducted, but a two-way table of regular tobacco use at baseline and at follow-up was presented, although only current snus use was reported. Among men who reported never smoking at baseline, the proportions reporting smoking at follow-up were 40/965 (4.1%) if they used snus at baseline and 51/3,596 (1.4%) if they did not do so, from which the RR can be estimated as 2.92 (95% CI 1.94–3.49). Among women, the corresponding proportions were 4/131 (3.1%) for snus users and 121/5,210 (2.3%), the RR being 1.31 (95% CI 0.49–3.51).

Two years later, in 2011, Norberg et al. [26] reported further results, this time based on 24,984 subjects examined initially in 1990–1997 and again 10 years later. Again, no specific test of the gateway hypothesis was included, and here the two-way table of tobacco use at baseline and at follow-up was only based on current habits at baseline and at follow-up. From the data presented, it was possible to calculate RRs for tobacco use at follow-up by snus use at baseline among those who were nonsmokers (not never smokers) at baseline. These RRs were estimated as 2.35 (95% CI 1.89–2.92) in men and as 2.53 (1.76–3.63) in women, somewhat higher than the corresponding estimates of 1.92 (1.43–2.57) for men and 1.43 (0.79–2.58) for women, which can be derived from the data presented in 2009 [25].

A major limitation of all these results is the lack of consideration of confounding variables. Also the results from the later paper based on nonsmokers at baseline will inappropriately include some subjects who will have smoked before first using snus.

### Other Swedish studies

The remaining studies in Sweden, discussed below, are of less direct use to testing the gateway hypothesis.

In 1990, in a very brief paper, Ramström [27] presented some results on the sequence of use of smoking and snus for men aged 18–34 years, based on the 1987 and 1988 Swedish National Smoking and Health Association (NTS) surveys. Of the total population, 39% had never used tobacco, 18% had used snus only, 21% had smoked only, 15% had used both, starting with smoking, and 7% had used both, starting with snus. While the proportion of men who eventually smoked is lower for those who started with snus (7/25 = 28%) than for those who did not start with snus (36/75 = 48%), this comparison is biased due to failure to adjust for the time available in which to start smoking, as illustrated in Table 1 and elsewhere [4]. While the authors concluded that “the occurrence of snuff dipping in this population of young men in Sweden has interacted with smoking habits in two ways, both to increase smoking in one subgroup [those who started with snus] and to decrease smoking in another subgroup [those who started with smoking and were more likely to quit than those who only smoked]”, no useful results to test the gateway hypothesis properly are actually presented.

Some results from these surveys are also reported in a paper the same year by Nordgren and Ramström [28], who commented that “taking up snuff must be seen as an introduction to the tobacco habit and possibly a first step towards taking up cigarettes”, there are again no results that can be used as an appropriate test of the gateway hypothesis. Further details on sequence of use are also available on the NTS in more extensive tabulations (e.g. [29]), but again they provide nothing of real value.

In 2003, Rolandsson and Hugoson [30] reported results of a study in which 183 ice hockey playing boys were interviewed in 1998 when aged 12–19 and then interviewed again in 2001. Although some information was presented on tobacco habits at baseline and follow-up and on take up of tobacco during the three year period, the data were not provided in a form that allowed initiation of smoking to be related to snus use at baseline. In any case, the number of smokers, 1 in 1998 and 13 in 2001, was very small. The authors discussed the gateway hypothesis, citing results from earlier studies, but did not draw conclusions from their own study.

In 2013, Rodu et al. [31] described results from the Northern Sweden multinational monitoring of trends and determinants in cardiovascular disease (MONICA) project comparing the joint prevalence of snus and smoking in random samples of men and women aged 25–64 interviewed in 1986, 1990, 1994, 1999, 2004 and 2009. Over the period, the prevalence of snus use tended to increase, while the prevalence of smoking clearly decreased; and the authors concluded that “use of snus was a significant factor in the low prevalence of smoking”. This study is not a proper test of the gateway hypothesis inasmuch as it provides no information on within-subject changes, the samples at the various time points being composed of different subjects. Also, other factors which may affect starting or quitting smoking, such as anti-smoking advice, are not considered.

### European smoking prevention framework approach (ESPAD) (Finland)

In 2006, Haukkala et al. [32] described results from the Finnish part of a study conducted in schools in six European countries. In half of the schools involved, pupils were subject to an intervention intended to affect “smoking-related attitudes, norms and self-efficacy cognitions”. Information on tobacco use was collected initially in September 1998 when the pupils started their seventh grade (T1) and then again in September 1999 (T2), in September 2000 (T3) and in April 2001 (T4). Among boys who were not regular smokers at T1, the OR for regular (at least weekly) smoking at T2 comparing those who had at least tried snus to those who had never tried was estimated as 6.21 (95% CI 3.20–12.06). Similarly, T2 snus experimentation predicted regular smoking at T3 (OR 4.38, 95% CI 2.82–6.80) while T3 snus experimentation predicted regular smoking at T4 (OR 4.37, 95% CI 2.44–7.82). The authors also presented results of a further analysis predicting regular smoking at T3 in nonsmokers at T2, in which school (experimental or control), sport participation and school achievement were included as predictors as well as snus use at T2. Here, the relationship to snus use was weakened, with ORs at 2.68 (95% CI 1.55–4.62) for a single try, 3.77 (2.09–6.78) for 2–50 tries, and 2.76 (1.26–6.06) for more than 50 tries. While noting the tendency for snus use to predict smoking in their analyses, the authors drew no clear conclusions regarding a causal relationship, noting the difficulties of controlling for the “common causes for all health-risk behaviours”.

Though the study fails to exclude former smokers from the at-risk population or present overall results over the whole follow-up period and adjusts for only a limited number of potential confounding variables, it is of some interest in demonstrating that the adjustment did in fact weaken the association of snus use to uptake of smoking.

### Oslo County and Hedmark study (Norway)

In 2013, Grøtvedt et al. [33] described results from a survey in which 1,395 male schoolchildren aged 16 at baseline in 2000–2001 were followed up 3 years later and answered the main tobacco questions on both occasions. The authors present a wide range of analyses relating tobacco habits at follow-up to those at baseline after adjustment for previous smoking, previous snus use, perceived family economy, alcohol use and first sexual experience by 10th grade, other variables (including age, parents’ marital status, country, parents’ country of birth, pupils’ educational plans and family members smoking) having been found not to independently influence the association between tobacco use at baseline and at follow-up. Although the authors noted that “Young men who only used snus at baseline had an increased risk of being dual users at follow-up” so that “Snus use may therefore facilitate smoking”, unfortunately none of the analyses presented were the most appropriate test of the gateway hypothesis, one problem being that they gave separate ORs for becoming smokers only and becoming dual users, not simply for becoming smokers.

Perhaps the most relevant results that they do present compare smoking habits at follow-up between those who at baseline were snus only users with those who were not tobacco users. Comparing current smoking only with no tobacco at follow-up, the unadjusted OR of 2.73 (95% CI 1.26–5.92) reduced to 1.66 (0.73–3.80) after adjustment; while comparing current dual users with no tobacco at follow-up, the unadjusted OR of 7.00 (3.78–12.96) reduced to 3.49 (1.79–6.82).

Though these analyses illustrate the considerable potential for confounding, highly unlikely to have been fully controlled for in these analyses, they are still not proper tests of the gateway hypothesis inasmuch as they concern current tobacco use at the time points considered rather than ever use. Also, the second comparison is severely biased, assuming independence of the two habits, as having both habits at follow-up is clearly going to be more likely if one has one habit to start with. Thus, if, for example, the probability of a non-user becoming a user over the interval is *p*_1_ for snus and *p*_2_ for smoking and these probabilities are assumed to be independent, it is apparent that the probability of a snus only user at baseline becoming a dual user at follow-up is *p*_1_ while the probability of a non tobacco user becoming a dual user is *p*_1_*p*_2_, which is clearly lower.

The source paper does provide a two-way table of tobacco use at baseline and at follow-up, from which it can be seen that the proportions smoking at follow-up were 35/90 (38.9%) among those who used snus only at baseline and 214/988 (21.7%) among those who used no tobacco at baseline. While this allows calculation of the RR as 1.79 (95% CI 1.25–2.57), this is not only unadjusted but relates only to current use at both time points.

### Annual nationally representative studies (Norway)

In 2014, Lund and Scheffels [34] described analyses involving 409 Norwegian men and women aged 15–74 years who had ever used snus and who had either never smoked or had started snus before cigarettes. The subjects were taken from annual cross-sectional surveys over the period 2005–2011, which had enquired about the age of initiation of tobacco use. The authors estimated the OR for ever having smoked in relation to starting snus before age 16 years as 2.75 (95% CI 1.76–4.31) after adjustment for gender and age at survey. Elevated adjusted odds ratios were also reported in analyses restricting attention to those who started snus at age 20 years or earlier (OR 2.24, 95% CI 1.42–3.53, *n* = 346 subjects) or those aged 30 years or younger at the time of the survey (2.18, 1.26–3.75, *n* = 250).

The very limited nature of the adjustment for potential confounding variables is clearly a major weakness of the study. Furthermore, even ignoring confounding, the method of analysis used is biased, since the time available to initiate smoking will differ according to whether snus was used or not. Inasmuch as data were available on time of starting to use snus and to smoke cigarettes, it would have been possible to use the methods described earlier to analyse the results of the study.

While the analyses add little to the evidence on the gateway hypothesis, it is of interest to note that though, in their sample, snus initiation occurred up to the age of 50 years, no one took up smoking after the age of 22 years.

In an earlier paper, in 2013, Lund and McNeill [35] also described results based on these repeated cross-sectional surveys but restricted to men. Though mainly concerned with dual use, they did report that only 24% of those with a history of dual use reported snus to be their first tobacco product, with the proportion varying by age, being 40.4% at age 15–24 years, 25.6% at age 25–44 years and 3.8% at age 45+ years. While the final percentage does indicate that snus use could hardly have been a major factor in the take up of smoking in the oldest age group, the results do not provide any proper test of the gateway hypothesis.

### Study of smoking cognitions (Norway)

Although not providing direct information on the relationship between prior snus use and initiation of smoking, a survey of Norwegian adolescents reported by Larsen et al. [36] deserves mention. In this study, questions were asked on two occasions 1 year apart. Sets of questions related to seven factors, “smoking cognitions”, that might be related to smoking initiation. There were no significant changes over the year in any of the smoking cognitions in the 160 regular snus users or in the 376 non-users of snus or cigarettes. However, in the 54 who had initiated snus in the period, a significant (*p* = 0.035) increase was noted in the score for the “negative affect reduction” factor, with the adolescents becoming more likely to answer positively on perceptions of smoking such as helping to calm down an angry person, to concentrate or to forget about problems at home. Although the change in answers to these questions suggested that snus use might facilitate smoking initiation, there was no evidence of any change in any of the other six cognitions that were studied (such as taste and sensorimotor stimulation, social facilitation and weight control). In view of the marginal *p* value for the single significant increase, the number of cognitions studied and the indirect nature of the evidence, this study provides no strong support for the gateway hypothesis.

## Discussion

In order to obtain a valid test of the gateway hypothesis, I have demonstrated that one should either use a cross-sectional study design in which data on time of starting to use snus and cigarettes (at some defined level) are obtained or a prospective cohort study design in which information is obtained at multiple time points on ever use of snus and cigarettes. Data should also be collected on potential confounding factors associated with initiation of smoking, such as age, sex, school achievement, alcohol use, drug use, readiness to take part in hazardous pursuits, delinquency, smoking by family members and friends and awareness of the hazards of smoking. One would then be able to carry out analyses relating smoking initiation to prior snus use, based on methods which allow for stratification by time of follow-up and adjustment for multiple potential confounders.

To what extent does the available published evidence on snus and smoking satisfy these ideal criteria? Before looking at this, it should be pointed out that though while for many of the studies considered, assessing the gateway hypothesis was clearly an objective in at least one of the publications, either being explicitly stated [18,27] or implicitly stated (e.g. by referring to the role of snus in the initiation of smoking) [20,22,24,32-34], assessment seemed not to be a major objective for quite a number [25,30,31]. It is unsurprising, therefore, that the ideal criteria are often not met.

The first criterion to consider is confounding, it being apparent that the level of adjustment is extremely unsatisfactory. Of the studies considered, many provide no adjusted results at all [18,19,25-31,35], while most of the remainder only consider simple demographic variables such as age and education [20,22-24,34]. Only two studies considered a longer list. One study in Finland [32] took sport participation and school achievement into account, as well as whether the school was subject to an intervention relating to attributes to smoking; while a study in Norway [33] took family economy, alcohol use and first sexual experience by 10th grade into account, other variables having been found not to independently affect the association between tobacco use at baseline and at follow-up. Only that study in Norway [33] can be regarded as having considered a reasonably complete list of potential confounding variables.

It is also apparent that the designs of many of the studies are seriously inadequate and could not possibly have allowed a proper investigation of the gateway hypothesis. Thus, one would reject the Swedish ULF [18,19] and the Finnish part of the ESPAD [32] studies because they only recorded data on current use, so not allowing study of smoking initiation in those who had never previously done so; the Swedish National NTS Surveys [27-29] because they only recorded information on sequence of snus use and smoking, and not on time of first use; the study in ice hockey players [30] because it is unrepresentative and based on a small sample; and the MONICA project [31] because there is no information on within-subject changes.

The remaining studies were of a design that made it possible to investigate the gateway hypothesis, though as noted virtually always failing to take potential confounding variables into account adequately. The SALT [22,23] and the Your Country and Your Life survey [24] were both cross-sectional surveys in which time of starting snus use and smoking was available but where the gateway hypothesis was studied using an invalid and extremely biased statistic, which failed to control for the time available for initiation. This same failure also applied to the analysis of the annual representative studies in Norway [34,35].

All these studies could have been analysed by time-stratified methods, but the published results give insufficient detail to allow this. The same is true for one of the three prospective studies, the BROMS survey of Swedish adolescents [20,21], which involved multiple assessments of smoking habits. The other two prospective studies, the VIP [25,26] and the Oslo County and Hedmark study of adolescents [33], involved assessment at two time points, so only allowing a simpler form of the analysis, based on the single follow-up. Only in the case of the earlier paper from the Västerbotten study [25] was it actually possible to derive a reasonably appropriate RR, though even here it was based on current rather than ever smoking at follow-up, and with no confounder adjustment.

Overall, 11 studies have been identified, of which five are of a design that does not allow proper testing of the gateway hypothesis. Of the remaining six, the analysis is unsatisfactory, sometimes seriously, in all of them. While it may be useful to revisit the databases for these six studies and attempt better analysis, it should be noted that only the Oslo County and Hedmark study [33] appears to have recorded data on an at all satisfactory set of confounding variables and then only recorded snus and smoking at two time points.

Given the abundant evidence that any direct health effects of snus are far less than those of smoking [4,37], snus use could only be regarded as unfavourable if it acted to encourage smoking initiation or discourage quitting. It seems a great pity, therefore that the first of these possibilities, the gateway hypothesis, has so far been so poorly addressed. There is a clear need for further studies. To obtain a rapid answer, a cross-sectional study in which data are recorded on time of first snus use and smoking and on a detailed list of factors that may affect initiation of smoking would be the obvious approach.

Attention has been limited specifically to investigating the hypothesis that snus use might act as a gateway to cigarette smoking. A related gateway is that between smokeless tobacco, as used in the United States, and smoking. Though the products typically used there differ from Swedish snus, it should be noted that there is quite an extensive literature on the subject (e.g. [12,38-42]), some of which pays detailed attention to confounding [12,41] as indicated earlier. However, it is beyond the scope of the present paper to evaluate how this literature stands up to the methods recommended above.

## Conclusions

There is currently no good information relating to the question of whether prior snus use might encourage initiation of smoking. All the studies have weaknesses in design or analysis that render their conclusions unreliable, particularly as data on other factors relevant to smoking initiation are not taken into account. Evidence is needed from better designed, better analysed studies which pay great attention to the problem of confounding.

## Abbreviations

A: the agent (e.g. snus), use of which may act as the gateway
B: the agent (e.g. smoking), use of which may be increased by use of A
BROMS: children’s smoking and environment in Stockholm County study
C: confounding variables
CI: confidence interval
ESPAD: European smoking prevention framework approach
MONICA: multinational monitoring of trends and determinants in cardiovascular disease
NTS: national smoking and health association study
OR: odds ratio
RR: relative risk
SALT: spanning across lifespan twin study
ULF: survey of living conditions
VIP: Västerbotten intervention programme
